# Dental Nurses’ Mental Health in Germany: A Nationwide Survey during the COVID-19 Pandemic

**DOI:** 10.3390/ijerph18158108

**Published:** 2021-07-30

**Authors:** Mohamed Mekhemar, Sameh Attia, Christof Dörfer, Jonas Conrad

**Affiliations:** 1 Clinic for Conservative Dentistry and Periodontology, School of Dental Medicine, Kiel University, Arnold-Heller-Str. 3, Haus B, 24105 Kiel, Germany; doerfer@konspar.uni-kiel.de; 2 Department of Oral and Maxillofacial Surgery, Justus-Liebig University Giessen, Klinik Str. 33, 35392 Giessen, Germany; sameh.attia@dentist.med.uni-giessen.de

**Keywords:** COVID-19, dental assistants, mental health, anxiety, depression, risk factors

## Abstract

Several studies have found a rise in the rate of psychological discomfort among healthcare personnel since the COVID-19 pandemic outbreak. In this study, we analyzed the relationship between psychological variables of anxiety, depression, stress, avoidance, intrusion and hyperarousal and several factors among German dental nurses. For this poll, dental nurses were asked nationwide to take part via an online-based survey from July 2020 to January 2021. This survey gathered data on demographics, as well as psychological assessments through the Impact of Events Scale-Revised (IES-R) instrument, and the Depression Anxiety Stress Scales (DASS-21). The correlations between DASS-21/IES-R ratings and sociodemographic data were investigated using univariate analyses (Kruskal–Wallis and Mann–Whitney U tests). Single comparisons were performed using the Dunn–Bonferroni post hoc test if a relevant test result was significant followed by multiple linear regressions. Furthermore, 252 dental nurses took part in the study and showed overall normal or mild results of all psychological variables. Having immune-deficiency or chronic diseases, employment at a dental practice, being married, having no children and seeing the pandemic as a financial threat were presented as significant risk factors (*p* ≤ 0.05) with higher DASS-21 and IES-R scores. These results emphasize the aspects that must be considered to safeguard German dental nurses’ mental wellbeing during the crisis.

## 1. Introduction

COVID-19, a novel infection, has been impacting public health and well-being worldwide since the year of 2020. The initial outbreak of the virus-related contagion was originally spotted in Wuhan, China and quickly spread throughout the globe. This increased disease transmission, combined with a growing number of infected cases and subsequent severe health issues or deaths, prompted intense public concern and fear. Early studies on the immediate mental effect of the infectious outbreak on the general population found that the outbreak had moderate to severe psychosomatic consequences [1].

An additional consequence of the outbreak on the population is the particular psychological burden of healthcare personnel, due to their close interaction with infected people and the augmented danger of disease transmission it entails [2]. These include concerns over infecting their families or loved ones with the disease [3], experiencing discrimination by the community as potential virus carriers [4], in addition to extensive workloads and time constraints, despite being critically understaffed or lacking the necessary safety measures [5]. This psychological load was observed worldwide among healthcare workers presenting high levels of anxiety, fear and depression with the nursing staff being the most psychologically hampered [6].

The COVID-19 pandemic stalled many efforts by healthcare workers, including professionals in the dental field [7,8,9]. Delaying routine care and using oral medicines had been postponed due to the amplified infection hazard when working on the mouth [10,11,12]. Furthermore, the oral mucosa has been recognized as a potential site of viral entrance [13]. To help decrease the danger of droplet infection, restricting dental treatments to just those treatments that are considered to be emergencies. A number of dental manufacturers, firms and clinics have let go some of their staff in order to manage the financial repercussions of the epidemic [14]. Previous research has established how dental practitioners view their moral obligation to restrict their daily work to prevent cross-infection amongst their loved ones and patients in spite of substantial financial concerns over a lockdown or decreased patient visits [15]. Other investigations declared the cessation of research or educational programs [15], an eventual emotional state of guilt among oral and dental and oral healthcare professionals, besides a lack of individual protective clinical equipment [16] as probable reasons of mental distress among oral-health and dental specialists during the global outbreak [15].

Since the beginning of SARS-CoV-2 cases in Germany, we have recorded over 3.65 million cases, with a high number of deaths related to COVID-19 health complications [17,18]. Federal states with the highest average incidence (CI cases per 100,000 residents) are Saxony (CI = 5845), Thuringia (CI = 4874), Bavaria (CI = 4090) and Berlin (CI = 4215) [19]. The pandemic’s distribution differs greatly across Germany and presents continuous dynamic transformation. Succeeding the first eruption of the disease in Germany by an infectious tourist [20], further cross-border transmissions were often triggered by people coming back from neighboring countries, while local hotspots of infection emerged in crowded activities such as concerts and carnivals [17]. Living conditions, such as clustered group accommodation and offices, have also been linked to high infection rates in the German population [17], or working situations close to infectious persons, such as in dental treatments [21].

Germany is considered a chief member within the European Union, with the largest number of residents and oral healthcare providers. About 80.5 million residents, almost 100,000 dentists, 200,000 dental nurses and 30 dental schools within Germany display the nation’s significant position as a healthcare center in Europe [22,23,24], in which oral healthcare procedures are offered to patients in dental practices or university clinics under a system of statutory or private health insurance [25]. Previous investigations among the dental society in Germany presented an overall mild or normal psychological burden experienced by German dentists [26] and dental students [27] during the health crisis. However, the psychological outcome of the outbreak and its related origins on dental nurses as crucial members of every dental society [28,29] remain unknown. As a result, this survey aimed to inspect this subject on a national scale among dental nurses in Germany using the Impact of Event Scale-Revised (IES-R) and Depression Anxiety Stress Scales (DASS-21) survey tools to specifically evaluate depression, anxiety and stress, as well as intrusion, avoidance and hyperarousal and their correlated risk factors amongst the investigation’s participants.

## 2. Materials and Methods

### 2.1. Study Population and Procedures

A nationwide cross-sectional survey was undertaken in Germany to determine the psychological effect of the present pandemic and its related causes on dental nurses. An online-based questionnaire was created using a digital survey platform to reduce physical contact and achieve more accessible involvement for all dental nurses (Unipark, QuestBack GmbH, Cologne, Germany). After receiving approval from the University of Kiel’s Ethics Board (D452/18), the survey link was distributed on many dental pages from various specializations on social networks, by many dental nurse blogs and websites, and dental publishers in Germany. A quick description of the study and the promise of anonymity and voluntary involvement for the participants were provided to kick-off the study. There were no financial incentives provided to participating nurses, and exclusion criteria were not implemented (gender, age or nationality). Prior to the commencement of the survey, all participants signed permission forms digitally stating their agreement to participate. The data collection period was between July 2020 and January 2021.

### 2.2. Survey Instruments

The first part of the questionnaire gathered sociodemographic data from participants such as their age, federal state, marital status, parenthood, smoking status and systemic comorbidities. Dental nurses also specified if they thought the outbreak represented a private financial risk.

Subsequent to that point, in the second part of the survey, respondents received the Depression Anxiety Stress Scale (DASS-21) and Impact of Event Scale-Revised (IES-R) tool. Both survey instruments were applied with their scoring systems and cut-off scores to evaluate depression, anxiety and stress, as well as intrusion, avoidance and hyperarousal as applied for German dentists and dental students previously [26,27]. The DASS-21 subscales were scored as follows: normal (0–4 DASS-21 points), mild (5–6 DASS-21 points), moderate (7–10 DASS-21 points), severe (11–13 DASS-21 points) and extremely severe (14+ DASS-21 points) for depression; normal (0–3 DASS-21 points), mild (4–5 DASS-21 points), moderate (6–7 DASS-21 points), severe (8–9 DASS-21 points) and extremely severe (10+ DASS-21 points) for anxiety; and normal (0–7 DASS-21 points), mild (8–9 DASS-21 points), moderate (10–12 DASS-21 points), severe (13–16 DASS-21 points) and extremely severe (17+ DASS-21 points) for stress. The IES-R subscores were categorized as normal (0–23 IES-R points), mild (24–32 IES-R points), moderate (33–36 IES-R points), and severe psychological impact of events (>37 IES-R points). Both survey instruments were validated before in their German version (Cronbach’s α 0.78–0.91) and displayed an important reliability to measure the psychological effects of the COVID-19 pandemic on general populations and healthcare workers [26].

### 2.3. Sample Size Calculation

The number of dental nurse participation needed for a national representative sample size was calculated using the following conditions:

1. The number of dental nurses in Germany (*N* = 200,000).
2. A 97% confidence level.
3. A 7% margin of error (MOE; calculated by MOE = z × √*p* × (1 − *p*)/√(*N* − 1) × *n*/(*N* − *n*)

It was estimated that on the basis of these circumstances, it was concluded that at least 240 dental nurses were required for a statistically significant sample size from all German federal states.

### 2.4. Statistical Analysis

The web-based survey tool digitally recorded the data from the online questionnaire, which was then exported for statistical analysis using SPSS software (SPSS Statistic 27, IBM, Armonk, NY, USA). Each query was given its own descriptive data analysis, and the Shapiro–Wilk test was used to test for the normality of the data. The data did not have a normal distribution. The correlations between DASS-21/IES-R ratings and sociodemographic data were investigated using univariate analyses (Kruskal–Wallis and Mann–Whitney U tests). Single comparisons were completed using the Dunn–Bonferroni post hoc test if a relevant test result was significant. The input of these previously identified, specific factors were then evaluated using multiple linear regression tests on the DASS-21 total and subscores and the IES-R subscales. Statistical significance was set at *p* ≤ 0.05.

## 3. Results

### 3.1. Sociodemographic Data of the Study Subjects

In this investigation, 252 dental nurses took part in the study with a bounce rate of nearly 55% resulting in a statistically significant sample. Corresponding sociodemographic data is presented in (Table 1).

### 3.2. DASS-21 and IES-R Scales and Risk Factors

Table 2 and Table 3 show the results of the psychological symptom analysis in the whole sample using the DASS-21 and IES-R scales, as well as the relevant components (Table 4 and Table 5).

The overall survey population had DASS-21 and IES-R scores indicating normal psychological behaviors with possible mild mental anxiety due to the pandemic, according to the applied scoring methodology (Table 2 and Table 3).

DASS-21 and IES-R scales assigned factors presented significantly higher scores, designating a higher psychological impact on subscale variables among participating dental nurses employed at private dental practices, having marriage or marriage-like relationships and no children, presenting systemic comorbid conditions, as well as amongst dental nurses considering the current pandemic to pose a financial risk (Table 4 and Table 5). 

Multiple regression analyses of DASS-21 total and subscores within the study model exhibited a significant effect of financial aspects, systemic immunodeficiency conditions and having children on the mental stress, depression and anxiety of German dental nurses (Table 6). Correspondingly, multiple regression analyses of IES-R scores significantly impacted equivalent factors besides pulmonary diseases on intrusion, avoidance and hyperarousal of the study subjects (Table 7).

## 4. Discussion

The initial COVID-19 patient was testified in Bavaria, Germany, in the first months of 2020 [20]. Rapid changes were started in the health sector, similar to worldwide reactions, and immediate efforts were taken to address the oncoming catastrophe. Nevertheless, this serious and exceptional situation had an unavoidable effect on health-care personnel around the country. Of all healthcare sectors, the dental field is primarily impacted by the viral spread in Germany and worldwide, owing to a number of factors affecting the mental well-being and financial safety of medical professionals throughout the pandemic and its related lockdowns [7,26,30,31]. As yet, this is the first investigation to examine the psychological effect of the outbreak on dental nurses in Germany on a nationwide scale.

In this study, 252 dental nurses participated digitally and finished the online survey, exhibiting a significant study sample size and representing Germany’s dental nurses in the investigation. Within the participations, more respondents answered the DASS-21 survey questions compared to the IES-R (Table 2), which might indicate a fatigue effect during online participation [32]. Furthermore, sociodemographic data of the participants presented an equal distribution of genders compared to the population of dental nurses in Germany (94–98% female) (Table 1) [33,34]. Furthermore, most survey participants were working in dental practices and were younger than 50 years old (Table 1). This resembles the described regular age and corresponding employment features stated previously among dental nurses in Germany [33]. Moreover, survey respondents displayed smoking of approximately 27% (Table 1), similar to reported results of German dental nurses [35], as well as the general German population (27%) [36].

Cardiovascular diseases displayed the highest rate of predominant systemic diseases amongst participating dental nurses, with nearly 11% (Table 1). This result conforms to the rates stated by previous reports on the German population (10–13%) [37] and German oral health professionals [26].

In the current investigation, German dental nurses presented no distressing or mild mental effects of COVID-19 as estimated by the DASS-21 and IES-R survey systems regarding stress, anxiety, depression, intrusion, avoidance and hyperarousal. This corresponds to the outcomes reported among German dentists [26] and dental students, [27] but strongly diverges from other countries reporting higher mental distress during the pandemic [11,30,38,39,40,41]. In fact, previous investigations observed the same outcome among non-dental healthcare professionals in Germany in comparison with other regions of the world [6], which may indicate the psychological importance of Germany’s effort in containing COVID-19 infection rates, stabilizing its population’s financial situation during the crisis and communicating the justification for its emergency policies [42,43,44]. In line with the previously mentioned high infection rates and COVID-19 cumulative incidence in various German federal states [45], several areas such as Thuringia, Bavaria and Berlin demonstrated higher psychological outcomes on DASS-21 and IES-R scales (Table 4 and Table 5). Furthermore, several related causes appear to play functional roles in the amount of stress, anxiety and depression, as well as possible post-traumatic stress disorder (PTSD) symptoms impacting German dental nurses throughout the pandemic. Unlike previous studies on oral healthcare employees and dentists, female contributors did not display significantly higher psychological distress scores (Table 4 and Table 5) compared to their male colleagues [26,30,46]. This could be due to the low number of males who participated in this investigation and are generally employed in the dental nursing profession in Germany. The current study further disclosed that being single, divorced or widowed and having children were correlated to lower total DASS-21 and IES-R scores and subscores than being married or in a marriage-like relationship (Table 4 and Table 5) as well as having no children. This observation was reported similarly among the German population during the pandemic. Individuals having no relationships showed significantly better mental health than married couples or participants living with a partner, particularly with a poor relationship quality [47]. Among various factors, the noticeable economic strain created by the COVID-19 crisis might be associated closely with a relationship deterioration of the affected individuals [48]. Moreover, previous investigations on the psychological well-being of families have described how parenthood is associated with enhanced mental health and stability [49,50]. As parenting and family relationships provide didactic coping and social sustenance as a shield against difficult situations, this process translates into decreased levels of mental distress [51,52].

During the COVID-19 pandemic, the existence of medical comorbidities has frequently been related to increased in-hospital complications and mortality rates [53,54]. Consistently, individuals with systemic disorders and medical risk factors showed higher levels of psychological distress [1,26]. In this study, German dental nurses have demonstrated the same significant effect throughout the pandemic among participants with systemic conditions of the respiratory and cardiovascular systems, or immune deficiency (Table 4 and Table 5). This result resembles previous outcomes of dentists and healthcare workers in Germany and other countries, where health personnel having immune deficiency diseases and chronic illnesses or living among individuals having these conditions significantly conveyed higher stress and anxiety scores [26,55,56]. Certainly, these medical conditions are described among the chief risk factors linked to complex medical complications after COVID-19 infection [57,58], which explains the observed psychological stress. Furthermore, COVID-19 infection can cause or worsen cardiovascular conditions such as myocardial infarction, arrhythmias and venous thromboembolism [59,60]. Additionally, hypertension was recognized as the most common comorbidity in COVID-19 patients [61], generating an evident stress aspect for cardiovascular patients [62]. Interestingly, although smoking is considered a chief risk factor for malignant and nonmalignant diseases affecting the respiratory system [63,64], participants of the current study did not show any significant differences in mental distress between smokers and non-smokers. This differs from similar investigations during the COVID-19 crisis but confirms the occasionally reported high percentage of smokers (50%) that showed no signs of stress throughout the outbreak [65,66,67,68].

Anxiety, stress related to workplace and depressive conditions are among the most prominent of many causes of psychological complications around the globe [69]. In previous studies, dentists and healthcare employees expressed numerous occupational stressors such as the infection risk, continuous-time pressure, anxiety about their ability to deliver sufficient health services in the future and financial burdens [26,70,71]. These occupational stressors dramatically increased among dental professionals worldwide during the COVID-19 pandemic [26,30,70]. Based on the findings of the current survey, dental nurses employed in private dental practices scored considerably higher on the DASS-21 and IES-R scales than their peers who work in university clinics (Table 4 and Table 5). Different aspects could clarify this observed result. While university clinics regularly have work-activities separated into three divisions comprising research, education and patient treatment, most private dental practices solely provide dental care services. This might provide dental nurses at universities with a relative sensation of protection from infectious exposure within ongoing patient treatment [26].

Moreover, previous observations pronounced the initial deficiency of protecting tools against the new infection in many non-university health facilities in Germany, increasing the hazard of cross-infection and associated mental distress [72]. Having to deal with multiple lockdowns and patient shortage, this situation shaped a new economic challenge for many dental practices and their employees in Germany, as well as to different non-university/governmental health institutions [72]. This economic crisis reflects one of the critical origins of mental problems among healthcare employees globally [73,74] and was confirmed by the current study. In this survey, respondents viewing the pandemic as a financial danger conveyed higher scores significantly on both DASS-21 and IES-R scales in all parameters (Table 4 and Table 5). Indeed, prior studies suggested that mitigation and suppression strategies, which are required to stop the spread, are likely to have an economic impact, with disastrous consequences for many small and medium-sized industries, including dental practices [75]. The longer these measures are continued, the more likely they seem to disturb the economic capacity of many dental practices and their employees, which may turn into job losses and further psychological distress [75].

Multiple analyses of linear regression were performed to determine the independent effects of the significant measured factors of COVID-19 being a financial threat, workplace, medical comorbidities and the relationship or family status on how the DASS-21 and IES-R scores and subscores performed. Being immune deficient, having chronic lung diseases, seeing the COVID-19 pandemic as a financial threat and not having children were independently related with worse psychological consequences (Table 4, Table 5, Table 6 and Table 7), screening these characteristics as the most effective on German dental nurses and their mental well-being throughout the COVID-19 pandemic.

## 5. Limitations

Up to the present time, this survey is the first one in Germany to inspect the psychological impact of COVID-19 on dental nurses nationwide. Nevertheless, we recognize several limitations to our investigation. First, the study is constrained by its cross-sectional structure and does not provide a longitudinal follow-up. Then again, misinterpretations in similar studies have been described to be equally distributed [26]. In this investigation, the detected outcomes of German dental nurses cannot be attributed entirely to the examined aspects and socio-environmental data. Unrecorded covariables and sociodemographic factors (including the exact financial difficulties and the number of patient treatments per day) could play an essential role in altering several outcomes or elucidations of the survey. Furthermore, the sample size of this survey is considered small with a relatively high margin of error. The data collection phase of this survey was finished within several months. The time sensitivity during the crisis, sudden fluctuations in regional regulations and constantly changing infection rates might also have impacted the outcomes reported by the participants.

Moreover, the voluntary nature of the study might have produced a selection bias amongst the participating nurses. Finally, to allow the maximum number of participations and reduce face-to-face circumstances, we used an online self-report survey to evaluate mental indications that do not depend on a diagnostic assessment by mental health specialists. Adding a clinical psychological analysis by psychiatric experts would undoubtedly contribute to the outcome of the investigation. Notwithstanding the mentioned limitations, deductions of this investigation deliver significant evidence on the mental outcome of the COVID-19 pandemic on dental nurses across Germany.

## 6. Conclusions

The mental health of dental nurses is critical for assuring the provision of dental services throughout the fight against the COVID-19 pandemic. The conclusions based on our survey population have shown that being chronically ill or immune deficient, working at a private dental practice, being married or in a similar relationship, as well as raising no children and perceiving the COVID-19 outbreak a personal financial risk are significant factors increasing psychological distress in German dental nurses during the current crisis with higher scores of DASS-21 and IES-R. Examining these features can support health and government authorities in Germany to implement the required arrangements to reduce the adverse mental outcomes of the pandemic and its associated aspects on the German dental society.

## Figures and Tables

**Table 1:** Participants’ characteristics (*n* = 252).

		*n*	%
**Gender**			
	Female	247	98.0
	Male	5	2.0
**Age**			
	18–49	193	76.5
	50–59	44	17.5
	≥60	15	6.0
**Marital Status**			
	Single	60	23.8
	Married or in a marriage-like partnership	168	66.7
	Divorced, separated or widowed	24	9.5
**Having Children**			
	Yes	138	54.8
	No	114	45.2
**COVID-19 Being a Personal Financial Threat**			
	Yes	138	54.8
	No	114	45.2
**Workplace ^1^**			
	Dental practice	219	86.9
	University clinic	20	7.9
	Other	13	5.2
**Federal State**			
	Hamburg	5	2.0
	Mecklenburg-Western Pomerania	7	2.8
	Schleswig-Holstein	43	17.1
	Brandenburg	3	1.2
	Berlin	11	4.4
	Lower Saxony	20	7.9
	Baden-Württemberg	61	24.2
	Thuringia	4	1.6
	Hesse	20	7.9
	Saarland	3	1.2
	Bavaria	17	6.7
	Saxony-Anhalt	1	0.1
	Saxony	11	4.4
	North Rhine-Westphalia	40	15.9
	Rhineland-Palatinate	6	2.4
**Smoker**			
	Yes	70	27.8
	No	182	72.2
**Medical Comorbidity ^2^**			
	Diseases of the cardiovascular system (e.g., coronary heart disease and high blood pressure)	28	11.1
	Chronic lung diseases (e.g., COPD)	9	3.6
	Chronic liver diseases	2	0.8
	Diabetes mellitus	12	4.8
	Cancer	2	0.8
	Immunodeficiency	15	6.0

^1^ Multiple choice was possible; ^2^ No or multiple choice was possible.

**Table 2:** DASS-21 and IES-R scores of the study subjects.

		Mean ± SD	Median	Interquartile Range
**DASS-21 (*n* = 252) ^1^**				
	Total	15.20 ± 13.44	11.00	20
	Depression	4.90 ± 4.98	3.00	7
	Anxiety	3.56 ± 4.29	2.00	6
	Stress	6.74 ± 5.19	6.00	8
**IES-R (*n* = 229) ^1^**				
	Intrusion	9.48 ± 9.02	7.00	14
	Avoidance	11.83 ± 9.34	10.00	14
	Hyperarousal	11.69 ± 9.78	9.00	16

DASS-21—Depression Anxiety Stress Scale; IES-R—Impact of Event Scale-Revised; ^1^ *n* varies because of missing data.

**Table 3:** Number of dental nurses and total population percentage for each DASS-21 and IES-R subscale category.

	Subscale	Category	*n*	%
**DASS-21 (*n* = 252) ^1^**				
	Depression	normal	142	56.3
		mild	32	12.7
		moderate	44	17.5
		severe	14	5.6
		extremely severe	20	7.9
	Anxiety	normal	161	63.9
		mild	27	10.7
		moderate	22	8.7
		severe	17	6.7
		extremely severe	25	9.9
	Stress	normal	146	57.9
		mild	34	13.5
		moderate	34	13.5
		severe	25	9.9
		extremely severe	13	5.2
**IES-R (*n* = 229) ^1^**				
	Intrusion	normal	209	91.3
		mild	17	7.4
		moderate	3	1.3
		severe	0	0
	Avoidance	normal	195	85.2
		mild	29	12.7
		moderate	4	1.7
		severe	1	0.4
	Hyperarousal	normal	194	84.7
		mild	29	12.7
		moderate	6	2.6
		severe	0	0

DASS-21—Depression Anxiety Stress Scale; IES-R—Impact of Event Scale-Revised; ^1^ *n* varies because of missing data.

**Table 4:** Differences in DASS-21 total and subscale scores based on individuals’ characteristics.

	DASS-21 Total			DASS-21 Depression			DASS-21 Anxiety			DASS-21 Stress		
	Mean ± SD	Test Statistic	*p*-Value	Mean ± SD	Test Statistic	*p*-Value	Mean ± SD	Test Statistic	*p*-Value	Mean ± SD	Test Statistic	*p*-Value
**Gender**												
Female	15.24 ± 13.53	U = 606.50	0.95	4.91 ± 5.02	U = 669.00	0.75	3.58 ± 4.31	U = 498.00	0.45	6.74 ± 5.21	U = 634.50	0.92
Male	13.20 ± 9.20			4.20 ± 2.17			2.40 ± 3.36			6.60 ± 4.51		
**Age**												
18–49	15.58 ± 13.50	H = 1.04	0.60	5.03 ± 4.97	H = 1.68	0.43	3.64 ± 4.35	H = 0.27	0.88	6.91 ± 5.20	H = 0.94	0.63
50–59	13.02 ± 11.51			4.00 ± 4.35			3.02 ± 3.56			6.00 ± 4.66		
≥60	16.67 ± 17.69			5.87 ± 6.61			4.07 ± 5.50			6.73 ± 6.48		
**Marital Status**												
Single	14.05 ± 12.34	H = 9.30	**0.01**	4.97 ± 4.60	H = 8.31	**0.02**	3.15 ± 3.86	H = 9.10	**0.01**	5.93 ± 5.06	H = 8.55	**0.01**
Married or in a marriage-like partnership	16.43 ± 13.72			5.15 ± 5.07			3.96 ± 4.47			7.33 ± 5.15		
Divorced, separated or widowed	9.42 ± 12.86			2.96 ± 5.03			1.79 ± 3.61			4.67 ± 5.19		
Post hoc: Dunn–Bonferroni test												
Divorced, separated or widowed-Single		35.81	**0.04**		45.05	**0.01**		30.91	0.07		20.88	0.24
Divorced, separated or widowed-Married or in a marriage-like partnership		47.68	**0.00**		44.66	**0.01**		45.27	**0.00**		40.86	**0.01**
Single-Married or in a marriage-like partnership		−11.87	0.84		0.39	0.97		−14.37	0.18		−19.98	0.07
**Having Children**												
Yes	14.01 ± 13.76	U = 9141.50	**0.03**	4.30 ± 4.87	U = 9385.50	**0.01**	3.33 ± 4.50	U = 8952.50	**0.05**	6.38 ± 5.23	U = 8683.50	0.16
No	16.64 ± 12.96			5.62 ± 5.02			3.83 ± 4.03			7.18 ± 5.11		
**COVID-19 Being a Personal Financial Threat**												
Yes	19.39 ± 14.21	U = 4556.00	**0.00**	6.36 ± 5.39	U = 4824.50	**0.00**	4.75 ± 4.79	U = 5060.00	**0.00**	8.28 ± 5.02	U = 4752.00	**0.00**
No	10.12 ± 10.44			3.12 ± 3.74			2.21 ± 3.06			4.88 ± 4.77		
**Workplace (Multiple Choice)**												
Dental practice	16.00 ± 13.76	U = 4550.50	**0.02**	5.17 ± 5.10	U = 4516.50	**0.02**	3.79 ± 4.43	U = 4462.50	**0.03**	7.03 ± 5.23	U = 4516.00	**0.02**
University clinic	9.15 ± 10.58	U = 1591.00	**0.02**	2.20 ± 3.33	U = 1398.50	**0.00**	2.20 ± 2.90	U = 1920.50	0.19	4.75 ± 5.15	U = 1703.00	**0.05**
other	11.08 ± 8.39	U = 1345.50	0.42	4.38 ± 3.69	U = 1572.00	0.94	1.77 ± 2.89	U = 1104.00	0.07	4.92 ± 3.52	U = 1268.00	0.26
**Federal state**												
Hamburg	11.00 ± 12.87	H = 27.55	**0.02**	3.00 ± 3.94	H =27.61	**0.02**	2.60 ± 3.58	H = 16.46	0.29	5.40 ± 5.94	H = 26.57	**0.02**
Mecklenburg-Western Pomerania	7.43 ± 15.36			2.57 ± 5.97			1.43 ± 3.36			3.43 ± 6.29		
Schleswig-Holstein	13.42 ± 12.42			4.30 ± 4.40			2.91 ± 3.86			6.21 ± 5.24		
Brandenburg	3.33 ± 4.93			0.33 ± 0.58			0.67 ± 1.16			2.23 ± 3.22		
Berlin	20.55 ± 14.95			6.73 ± 5.57			4.64 ± 5.05			9.18 ± 5.08		
Lower Saxony	15.45 ± 11.17			3.65 ± 3.07			4.05 ± 4.08			7.75 ± 5.31		
Baden-Württemberg	17.80 ± 14.54			5.98 ± 5.64			4.07 ± 4.71			7.75 ± 5.23		
Thuringia	23.00 ± 13.04			8.00 ± 4.97			6.00 ± 4.08			9.00 ± 4.08		
Hesse	12.75 ± 12.81			4.45 ± 5.31			2.65 ± 3.31			5.65 ± 4.84		
Saarland	19.67 ± 27.30			7.00 ± 9.64			6.00 ± 10.39			6.67 ± 7.37		
Bavaria	15.53 ± 16.74			5.18 ± 6.34			4.00 ± 5.27			6.35 ± 5.70		
Saxony-Anhalt	20.00 ± 0.00			4.00 ± 0.00			9.00 ± 0.00			7.00 ± 0.00		
Saxony	5.36 ± 5.89			1.36 ± 1.75			1.73 ± 2.28			2.27 ± 2.37		
North Rhine-Westphalia	16.43 ± 11.86			5.50 ± 4.33			3.83 ± 4.26			7.10 ± 4.59		
Rhineland-Palatinate	18.17 ± 11.05			5.68 ± 2.94			3.83 ± 3.19			8.67 ± 5.61		
**Smoker**												
Yes	14.70 ± 12.49	U = 6369.00	0.99	4.50 ± 4.59	U = 6632.00	0.61	3.51 ± 4.04	U = 6116.00	0.62	6.70 ± 4.96	U = 6350.00	0.97
No	15.39 ± 13.82			5.05 ± 5.12			3.58 ± 4.40			6.76 ± 5.28		
**Medical Comorbidity (Multiple Choice)**												
No medical comorbidities	14.87 ± 13.32			4.82 ± 5.01			3.36 ± 4.14			6.69 ± 5.13		
Diseases of the cardiovascular system (e.g., coronary heart disease and high blood pressure)	12.61 ± 14.53	U = 2594.00	0.14	4.18 ± 5.14	U = 2808.50	0.36	3.25 ± 5.15	U = 2699.50	0.22	5.18 ± 5.12	U = 2470.50	0.07
Chronic lung diseases (e.g., COPD)	21.33 ± 17.83	U = 1348.50	0.24	6.44 ± 6.25	U = 1289.50	0.36	6.56 ±5.90	U = 1460.50	0.08	8.33 ± 5.98	U = 1287.00	0.90
Chronic liver diseases	15.00 ± 21.21	U = 226.50	0.82	2.50 ± 3.54	U = 177.50	0.48	4.50 ± 6.36	U = 261.50	0.91	8.00 ± 11.31	U = 249.00	0.99
Diabetes mellitus	16.42 ± 13.60	U = 1531.50	0.71	4.92 ± 4.38	U = 1491.50	0.83	3.83 ± 4.22	U = 1541.00	0.68	7.67 ± 6.02	U = 1569.50	0.60
Cancer	15.50 ± 6.36	U = 295.00	0.66	3.50 ± 4.95	U = 205.50	0.66	2.00 ± 0.00	U = 242.00	0.94	10.00 ± 1.41	U = 367.00	0.25
Immunodeficiency	22.53 ± 12.98	U = 2433.00	**0.02**	7.40 ± 4.85	U = 2404.00	**0.02**	6.27 ± 4.30	U = 2552.00	**0.00**	8.87 ± 5.22	U = 2243.00	0.09

DASS-21 = Depression Anxiety Stress Scale; SD = standard deviation; H = test statistic of Kruskal–Wallis test; U = test statistic of Mann–Whitney U test; significant results (*p* ≤ 0.05) are bolded.

**Table 5:** Differences in IES-R subscale scores based on participant characteristics.

	IES-R Intrusion			IES-R Avoidance			IES-R Hyperarousal		
	Mean ± SD	Test Statistic	*p*-Value	Mean ± SD	Test Statistic	*p*-Value	Mean ± SD	Test Statistic	*p*-Value
**Gender**									
Female	9.54 ± 9.09	U = 508.00	0.72	11.92 ± 9.40	U = 457.50	0.48	11.72 ± 9.87	U = 577.00	0.91
Male	6.40 ± 4.62			8.20 ± 5.07			10.40 ± 5.03		
**Age**									
18–49	9.27 ± 9.21	H = 0.93	0.63	12.36 ± 9.72	H = 1.86	0.40	11.74 ± 9.85	H = 0.26	0.88
50–59	10.11 ± 8.56			10.55 ± 7.84			11.21 ± 9.62		
≥60	10.27 ± 8.24			8.87 ± 7.55			12.33 ± 10.00		
**Marital Status**									
Single	9.30 ± 8.42	H = 4.29	0.12	13.05 ± 10.40	H = 3.40	0.18	10.86 ± 9.24	H = 6.71	**0.04**
Married or in a marriage-like partnership	9.99 ± 9.39			11.82 ± 9.00			12.61 ± 10.03		
Divorced, separated, or widowed	6.05 ± 7.23			8.50 ± 8.22			7.15 ± 8.23		
Post hoc: Dunn–Bonferroni-Test									
Divorced, separated, or widowed-Single								30.20	0.08
Divorced, separated, or widowed-Married or in a marriage-like partnership								39.97	**0.01**
Single-Married or in a marriage-like partnership								−9.78	0.34
**Having Children**									
Yes	9.07 ± 9.54	U = 7203.00	0.16	10.44 ± 9.02	U = 7840.50	**0.01**	10.74 ± 9.94	U = 7499.00	**0.05**
No	9.95 ± 8.40			13.49 ± 9.47			12.82 ± 9.51		
**COVID-19 Being a Personal Financial Threat**									
Yes	12.16 ± 9.47	U = 3748.00	**0.00**	14.02 ± 9.21	U = 4250.00	**0.00**	14.67 ± 9.88	U = 3802.50	**0.00**
No	6.08 ± 7.13			9.07 ± 8.78			7.92 ± 8.28		
**Workplace (Multiple Choice)**									
Dental practice	9.91 ± 9.31	U = 3631.00	0.17	12.34 ± 9.42	U = 3883.00	**0.04**	12.29 ± 10.00	U = 3875.00	**0.04**
University clinic	6.26 ± 6.05	U = 1649.00	0.21	6.26 ± 4.63	U = 1272.00	**0.01**	7.74 ± 7.21	U = 1551.50	0.11
Other	7.62 ± 7.18	U = 1271.00	0.57	12.31 ± 10.99	U = 1396.00	0.97	8.38 ± 7.97	U = 1124.50	0.23
**Federal State**									
Hamburg	3.80± 7.43	H = 33.25	**0.00**	7.20 ± 10.89	H = 35.38	**0.00**	7.00 ± 10.46	H = 36.61	**0.00**
Mecklenburg-Western Pomerania	2.00 ± 4.47			3.14 ± 5.64			3.43 ± 6.80		
Schleswig-Holstein	8.34 ± 8.25			9.63 ± 7.78			9.89 ± 9.33		
Brandenburg	1.50 ± 2.12			3.50 ± 2.12			1.50 ± 2.12		
Berlin	14.89 ± 8.67			14.00 ± 9.61			17.33 ± 11.23		
Lower Saxony	10.40 ± 9.80			15.80 ±9.48			13.60 ± 9.23		
Baden-Württemberg	11.20 ± 9.88			14.29 ± 10.29			14.43 ± 9.71		
Thuringia	15.75 ± 9.00			17.00 ± 10.13			15.75 ± 9.50		
Hesse	8.21 ± 7.64			8.47 ± 7.18			8.74 ± 9.63		
Saarland	7.33 ± 11.02			5.00 ± 5.00			9.67 ± 13.32		
Bavaria	9.87 ± 9.63			12.87 ± 11.40			11.60 ± 11.62		
Saxony-Anhalt	18.00 ± 0.00			14.00 ± 0.00			19.00 ± 0.00		
Saxony	2.36 ± 2.80			5.91 ± 4.01			4.09 ± 6.75		
North Rhine-Westphalia	10.29 ± 8.97			12.79 ± 8.77			12.84 ± 8.65		
Rhineland-Palatinate	12.67 ± 8.85			19.00 ± 6.93			14.67 ± 8.57		
**Smoker**									
Yes	8.41 ± 7.52	U = 5162.00	0.64	12.31 ± 9.11	U = 4681.00	0.52	10.67 ± 8.66	U = 5237.00	0.52
No	9.84 ± 9.47			11.67 ± 9.43			12.04 ± 10.14		
**Medical Comorbidity (Multiple Choice)**									
No medical comorbidities	9.14 ± 8.91			11.53 ± 8.92			11.07 ± 9.52		
Diseases of the cardiovascular system (e.g., coronary heart disease and high blood pressure)	8.81 ± 9.96	U = 2431.00	0.51	8.96 ± 9.61	U = 1995.00	**0.04**	10.88 ± 11.00	U = 2423.50	0.50
Chronic lung diseases (e.g., COPD)	14.63 ± 13.32	U = 1077.00	0.29	19.88 ± 12.47	U = 1239.00	**0.05**	14.00 ± 11.69	U = 975.00	0.62
Chronic liver diseases	1.50 ± 2.12	U = 94.00	0.15	6.50 ± 4.95	U = 156.00	0.45	9.50 ± 6.36	U = 215.50	0.90
Diabetes mellitus	13.67 ± 10.99	U = 1590.50	0.20	14.17 ± 11.41	U = 1443.00	0.53	15.50 ± 11.61	U = 1536.50	0.29
Cancer	7.00 ± 0.00	U = 115.00	0.99	5.00 ± 0.00	U = 63.00	0.44	14.00 ± 0.00	U = 144.50	0.64
Immunodeficiency	13.21 ± 8.20	U = 1930.00	0.08	18.64 ± 10.10	U = 2141.00	**0.01**	19.14 ± 9.37	U = 2188.00	**0.00**

IES-R = Impact of Event Scale-Revised; SD = standard deviation; H = test statistic of Kruskal–Wallis test; U = test statistic of Mann–Whitney U test; significant results (*p* ≤ 0.05) are bolded.

**Table 6:** Multiple regression studies of DASS-21 total and subscores with pertinent variables.

	B	SE	Β	T	p	95% CI
**DASS-21 Total**						
Marital status ^a^	0.34	1.65	0.01	0.20	0.84	−2.91; 3.58
Having children ^b^	3.64	1.85	0.14	1.97	**0.05**	−0.00; 7.28
COVID-19 being personal financial threat ^b^	−9.13	1.63	0.34	−5.59	**0.00**	−12.35; −5.91
Workplace: Dental practice ^c^	2.86	3.65	0.07	0.78	0.43	−4.32; 10.03
Workplace: University clinic ^c^	−1.33	4.54	0.03	−0.29	0.77	−10.27; 7.62
Federal state ^d^	0.02	0.17	0.01	0.14	0.89	−0.32; 0.36
Medical comorbidity: Immunodeficiency ^c^	7.22	3.35	0.13	2.16	**0.03**	0.62; 13.81
**DASS-21 Depression**						
Marital status ^a^	−0.06	0.61	−0.01	−0.09	0.93	−1.26; 1.15
Having children ^b^	1.54	0.69	0.16	2.25	**0.03**	0.19; 2.89
COVID-19 being personal financial threat ^b^	−3.23	0.61	−0.32	−5.34	**0.00**	−4.43; −2.04
Workplace: Dental practice ^c^	0.16	1.35	0.01	0.12	0.90	−2.50; 2.82
Workplace: University clinic ^c^	−1.84	1.68	0.10	−1.10	0.27	−5.16; 1.47
Federal state ^d^	0.01	0.06	0.01	0.16	0.88	−0.12; 0.14
Medical comorbidity: Immunodeficiency ^c^	2.42	1.24	0.12	1.95	**0.05**	−0.03; 4.86
**DASS-21 Anxiety**						
Marital status ^a^	−0.02	0.53	−0.00	−0.03	0.98	−1.06; 1.03
Having children ^b^	0.71	0.60	0.08	1.19	0.24	−0.47; 1.89
COVID-19 being personal financial threat ^b^	−2.58	0.52	−0.30	−4.91	**0.00**	−3.61; −1.54
Workplace: Dental practice ^c^	1.02	0.77	0.08	1.32	0.19	−0.50; 2.54
Medical comorbidity: Immunodeficiency ^c^	2.73	1.08	0.15	2.53	**0.01**	0.60; 4.87
**DASS-21 Stress**						
Marital status ^a^	−0.44	0.47	−0.06	−0.93	0.35	−1.36; 0.49
COVID-19 being personal financial threat ^b^	−2.51	0.54	−0.29	−4.67	**0.00**	−3.56; −1.45
Workplace: Dental practice ^c^	1.46	1.19	0.12	1.23	0.22	−0.89; 3.80
Workplace: University clinic ^c^	0.71	1.49	0.05	0.47	0.64	−2.23; 3.64
Federal state ^d^	0.03	0.06	0.03	0.47	0.64	−0.09; 0.14

B = unstandardized beta coefficient; SE = standard error; β = standardized beta coefficient; p = *p*-value; CI: confidence interval; significant results (*p* ≤ 0.05) are bolded; ^a^ 1 = Single; 2 = Married or in a marriage-like partnership; 3 = Divorced, separated or widowed; ^b^ 1 = yes; 2 = no; ^c^ 0 = not quoted; 1 = quoted; ^d^ 5 = Bremen; 6 = Hamburg; 8 = Mecklenburg-Western Pomerania; 15 = Schleswig-Holstein; 16 = Brandenburg; 17 = Berlin; 18 = Lower Saxony; 19 = Baden-Württemberg; 20 = Thuringia; 21 = Hesse; 22 = Saarland; 23 = Bavaria; 24 = Saxony-Anhalt; 25 = Saxony; 26 = North Rhine-Westphalia; 27 = Rhineland-Palatinate.

**Table 7:** Multiple regression studies of IES-R scores with pertinent covariates.

	B	SE	β	T	p	95% CI
**IES-R Intrusion**						
COVID-19 being personal financial threat ^b^	−5.94	1.15	−0.33	−5.19	**0.00**	−8.20; −3.69
Federal state ^d^	0.10	0.12	0.05	0.83	0.41	−0.14; 0.33
**IES-R Avoidance**						
Having children ^b^	3.29	1.16	0.18	2.83	**0.01**	0.99; 5.58
COVID-19 being personal financial threat ^b^	−4.64	1.19	−0.25	−3.90	**0.00**	−6.98; −2.29
Workplace: Dental practice ^c^	−1.24	2.57	−0.05	−0.48	0.63	−6.30; 3.82
Workplace: University clinic ^c^	−5.39	3.19	−0.16	−1.69	0.09	−11.68; 0.90
Federal state ^d^	0.08	0.12	0.04	0.60	0.55	−0.17; 0.32
Medical comorbidity: Diseases of the cardiovascular system (e.g., coronary heart disease and high blood pressure) ^c^	−2.69	1.85	−0.09	−1.46	0.15	−6.33; 0.95
Medical comorbidity: Chronic lung diseases (e.g., COPD) ^c^	7.24	3.20	0.14	2.27	**0.02**	0.94; 13.53
Medical comorbidity: Immunodeficiency ^c^	6.29	2.40	0.16	2.62	**0.01**	1.57;11.02
**IES-R Hyperarousal**						
Marital status ^a^	0.23	1.25	0.01	0.18	0.86	−2.23; 2.68
Having children ^b^	2.58	1.38	0.13	1.26	0.06	−0.15; 5.31
COVID-19 being personal financial threat ^b^	−6.67	1.24	−0.34	−5.37	**0.00**	−9.12; −4.22
Workplace: Dental practice ^c^	2.31	1.75	.08	1.32	0.19	−1.15; 5.77
Federal state ^d^	0.07	0.13	0.04	0.56	0.58	−0.18; 0.32
Medical comorbidity: Immunodeficiency ^c^	7.70	2.49	0.19	3.09	**0.00**	2.80; 12.61

B = unstandardized beta coefficient; SE = standard error; β = standardized beta coefficient; p = *p*-value; CI: confidence interval; significant results *(p* ≤ 0.05) are bolded;.^a^ 1 = Single; 2 = Married or in a marriage-like partnership; 3 = Divorced, separated or widowed; ^b^ 1 = yes; 2 = no; ^c^ 0 = not quoted; 1 = quoted; ^d^ 5 = Bremen; 6 = Hamburg; 8 = Mecklenburg-Western Pomerania; 15 = Schleswig-Holstein; 16 = Brandenburg; 17 = Berlin; 18 = Lower Saxony; 19 = Baden-Württemberg; 20 = Thuringia; 21 = Hesse; 22 = Saarland; 23 = Bavaria; 24 = Saxony-Anhalt; 25 = Saxony; 26 = North Rhine-Westphalia; 27 = Rhineland-Palatinate.

## Data Availability

The data presented in this study are available on request from the corresponding author.
